# Determining the diagnostic cut-off on the Chinese version of severity of dependence scale for cannabis

**DOI:** 10.3389/fpsyt.2024.1495119

**Published:** 2025-01-07

**Authors:** Albert Kar Kin Chung, Cheuk Yin Tse

**Affiliations:** Department of Psychiatry, Li Ka Shing Faculty of Medicine, The University of Hong Kong, Hong Kong SAR, China

**Keywords:** cannabis, Chinese SDS, screening, cannabis use disorder, psychometrics

## Abstract

**Introduction:**

Cannabis use and misuse are surging among the Chinese community in East and Southeast Asia. A quick screening instrument that can effectively identify users with dependence for early intervention is in utmost need. This study examined the psychometric properties of the Chinese version of the Severity of Dependence Scale for cannabis (C-SDS-C) in screening for the DSM-5 defined Cannabis Use Disorder (CUD).

**Methods:**

A retrospective chart review was conducted on Chinese-speaking individuals reporting cannabis use from three different substance use studies. Their demographic data, frequency of cannabis use within the past 30 days, scorings for the C-SDS-C and the severity of CUD at baseline were analyzed.

**Results:**

The C-SDS-C exhibited high reliability (Cronbach’s alpha = 0.778). It had a strong correlation with the severity of CUD (r = 0.456, *p* <.001) and a moderate correlation with the frequency of cannabis use within the past 30 days (r = 0.335, *p* = .001). All items loaded into a single factor which accounted for 56.64% of the variance. Receiver operating characteristic analysis demonstrated that a C-SDS-C cut-off score of ≥ 3 provided optimal discrimination for moderate to severe CUD among Chinese-speaking individuals using cannabis.

**Conclusion:**

The C-SDS-C is a valid and reliable screening instrument to identify cannabis users with moderate-to-severe CUD in the Chinese-speaking population.

## 1 Introduction

Cannabis has become the most widely used substance worldwide (1). The amount of cannabis herbs seized in East and Southeast Asia recorded a quadruple growth from 2011 to 2021 (2). It also ranks the third most prevalent drug for which individuals seek treatment in this region (3). In Hong Kong, data from the Narcotics Division indicate that the number of cannabis users has doubled from 2019 to 2023, with more than half of these users being under 21 years of age (4). Similar uprising trends are observed in other Chinese-speaking regions, including mainland China (5), Taiwan (6), and Malaysia (7). Notably, the lifetime prevalence of cannabis use in Singapore is the highest among all illicit drugs (8). Individuals diagnosed with cannabis use disorder (CUD) suffer a high prevalence of depressive and anxiety disorders (9–13) and are at a significant greater risk of developing psychotic disorders compared to non-users (11). Furthermore, cannabis use is associated with higher risks of self-harm and suicidal ideation (12, 13). Given that 17% of the total world population speaks Chinese as their native language (14, 15), it is imperative to have a validated screening tool in Chinese to identify Chinese-speaking cannabis users showing symptoms of CUD as early as possible for timely intervention.

The Severity of Dependence Scale (SDS) is a highly effective assessment tool for diagnosing drug dependence. This five-item, self-administered scale measures the psychological dependence experienced by drug users (16, 17). These items evaluate concerns related to impaired control, and the preoccupation and anxiety about drug use among people who use drugs. The SDS has exhibited strong validity through its correlations to both the dosage and frequency of various drugs, including heroin, cocaine, amphetamine, cannabis and benzodiazepine (17–21). It has also demonstrated good internal consistency with a single factor structure and high test-retest reliability (14, 21). Previously, different diagnostic cut-off scores on the SDS have been proposed for various drugs to predict drug dependence in accordance with the Diagnostic and Statistical Manual of Mental Disorders (DSM) (18–20, 22–24).

The original English version of SDS has been translated into multiple languages and is utilized in numerous countries (25–30). Despite the successful adaptation of the Chinese version of the SDS (C-SDS) in China and in Hong Kong, previous studies have primarily validated its use for dependence of heroin, benzodiazepines, ketamine, and the broad term of “club drugs” (26, 31). To date, no research has established its psychometric properties specifically for cannabis use.

The current study aims to investigate the diagnostic cut-off for the C-SDS in screening CUD for individuals using cannabis, in particularly moderate and severe CUD which indicates cannabis dependence. A diagnostic cut-off was determined using receiver operating characteristic (ROC) analysis to provide the optimal discrimination for at least moderate CUD. This would allow the C-SDS for cannabis (C-SDS-C) to serve as a rapid screening tool in the daily clinical practice.

## 2 Methods

### 2.1 Participants

A retrospective data review was conducted on three studies formerly approved by the Institutional Review Board of the University of Hong Kong/Hospital Authority Hong Kong West Cluster (HKU/HA HKW IRB): two on cannabis (UW 18-095 and UW 20-189) and one on commonly misused drugs including cannabis (UW 19-228), spanning from August 2018 to October 2023. All three studies were conducted in accordance with the Declaration of Helsinki. All subjects in these studies primarily spoke Chinese as their mother tongue. They were recruited through random sampling from substance misuse treatment centers and the community. Subjects who reported using cannabis for at least six times in the previous six months or had at least two positive urine tests for cannabis within the 30 days prior to enrollment, and who had completed the C-SDS and the Structured Clinical Interview for DSM-5 (SCID-5) at baseline, were identified from the study records for inclusion in the current study. Cannabis user diagnosed with intellectual disabilities, psychosis, or mood disorders were excluded from this study.

### 2.2 Design

For subjects meeting the inclusion criteria, data on demographics, frequency of cannabis use within the past 30 days, scorings on C-SDS, and the severity of CUD as verified by board-certified psychiatrists using (SCID-5), were retrieved from their records.

Subjects reporting cannabis use in the three included studies completed the same C-SDS, which has been previously translated into traditional Chinese and validated by Tung et al. (2014) for ketamine users. The C-SDS consisted of the same five items as the English version of SDS: (1) “Did you think your use of cannabis was out of control?”; (2) “Did the prospect of missing a smoke of cannabis make you anxious or worried?”; (3) “Did you worry about your use of cannabis?”; (4) “Did you wish you could stop?”; and (5) “How difficult did you find to stop or go without cannabis?”. Each item anchored on a 4-point scale. Participants scored the first four items among “0: never/almost never”, “1: sometimes”, “2: often”, and “3: always/nearly always”; while the last item among “0: not difficult”, “1: quite difficult”, “2: very difficult” and “3: impossible”. The total SDS score ranges from 0 to 15, with higher scoring above the drug-specific cut-off scores indicating greater dependence. As specified in the DSM-5, mild CUD and moderate-to-severe CUD correspond to cannabis harmful use and cannabis dependence syndrome, respectively, as defined in the Tenth Edition of International Classification of Diseases Clinical Modification (ICD-10-CM).

This study was approved by the HKU/HA HKW IRB (UW 23-267) and was not sponsored by any external funding sources.

### 2.3 Statistical analysis

Demographic data, mean C-SDS scores for different severities of CUD, and the frequency of cannabis use in the past 30 days were presented with descriptive statistics. The reliability of the C-SDS-C was assessed using Cronbach’s alpha. Correlations between C-SDS-C scores and both the severity of CUD and the frequency of cannabis use in the past 30 days were examined to evaluate its concurrent validity. Factor analysis using principal component analysis (PCA) was conducted to investigate the construct validity of C-SDS-C. Lastly, receiver operating characteristic (ROC) analysis was performed to determine the area under curve (AUC) and diagnostic cut-offs on C-SDS-C for mild CUD (cannabis harmful use) and moderate-to-severe CUD (cannabis dependence syndrome). All analyses were conducted using IBM SPSS Statistics for Windows, Version 29.0, with a significance of alpha = .05.

## 3 Results

### 3.1 Characteristics of participants

From the 441 records reviewed across the three studies, 90 subjects (20%) were identified as single drug users with cannabis who fulfilled the inclusion and exclusion criteria. Among these individuals, the majority were male (71.1%), with a mean age of 33.62 years old (SD = 11.8) (**Table 1**). The mean C-SDS-C score for all cannabis users was 3.72 (SD = 3.17), and the average frequency of cannabis use within the past 30 days was 9.42 days (SD = 11.37). Of these cannabis users, 21 (23.3%) did not meet the diagnostic criteria of CUD. A total of 33 users (36.7%) were diagnosed with mild CUD, while 36 users (40%) were classified as having moderate-to-severe CUD. Those with moderate-to-severe CUD were older (M = 29.25 *vs* M = 36.12), had a higher proportion of male users (75% *vs* 57.6%), scored higher on the C-SDS-C (M = 5.36 *vs* M = 3.03), and reported more frequent cannabis use within the past 30 days (M = 12.67 *vs* M = 8.68) relative to users with mild CUD.

**Table 1:** Demographics, C-SDS scores and frequency of cannabis use for non-CUD and CUD participants (N=90).

	No CUD (N=21)	Mild CUD (Harmful Use) (N=33)	Moderate to Severe CUD (Dependence Syndrome) (N=36)
Age, Mean (Range)	37.19 (47)	36.12 (41)	29.25 (36) Moderate: 26.58 (27) Severe: 32.24 (36)
Male Gender, N (%)	18 (85.7)	19 (57.6)	27 (75) Moderate: 14 (73.7) Severe: 13 (76.5)
C-SDS-C scores, Mean (SD)	2 (1.924)	3.03 (2.963)	5.36 (3.22) Moderate: 4.53 (2.27) Severe: 6.29 (3.89)
Frequency of cannabis use in the past 30 days, Mean (SD)	5.19 (9.21)	8.58 (11.64)	12.67 (11.58) Moderate: 11.74 (11.98) Severe: 13.71 (11.39)

C-SDS, Chinese version of the Severity of Dependence Scale; CUD, cannabis use disorder; N, number of participants; SD, standard deviation.

### 3.2 Validity, reliability, and factor analysis

C-SDS-C demonstrated a strong correlation with the severity of CUD (r = 0.456, *p* <.001) and a moderate correlation with the frequency of cannabis use within the past 30 days (r = 0.335, *p* = .001). The C-SDS-C scores mounted up alongside with the severity of CUD and the frequency of cannabis use, suggesting high concurrent validity. The internal consistency of the C-SDS-C measured by the Cronbach’s alpha was 0.778. Regarding the factor analysis, the Kaiser-Meyer-Olkin measure of sampling adequacy for the C-SDS-C was 0.76. The PCA extracted a single factor, accounting for 56.64% of the variance (**Table 2**). Except for Item 4, which had a weak factor loading (= .555), all other four items possessed strong factor loading characteristics (> 0.6) (**Table 3**). The reliability of C-SDS-C would improve further if Item 4 was removed (Cronbach’s alpha = 0.82).

**Table 2:** Principal component analysis of C-SDS-C.

Factor	Eigenvalues	% of Variance explained
1	2.83	56.64
2	0.79	15.82
3	0.68	13.49
4	0.48	9.51
5	0.23	4.54

C-SDS-C, Chinese version of the Severity of Dependence Scale for Cannabis.

**Table 3:** Factor loadings for each item in C-SDS-C.

Item	Factor loadings
1. Did you think your use of cannabis was out of control?	0.83
2. Did the prospect of missing a smoke of cannabis make you anxious or worried?	0.78
3. Did you worry about your use of cannabis?	0.87
4. Did you wish you could stop?	0.56
5. How difficult did you find it to stop or go without cannabis?	0.69

C-SDS-C, Chinese version of the Severity of Dependence Scale for Cannabis.

### 3.3 ROC analysis

**Figure 1:**
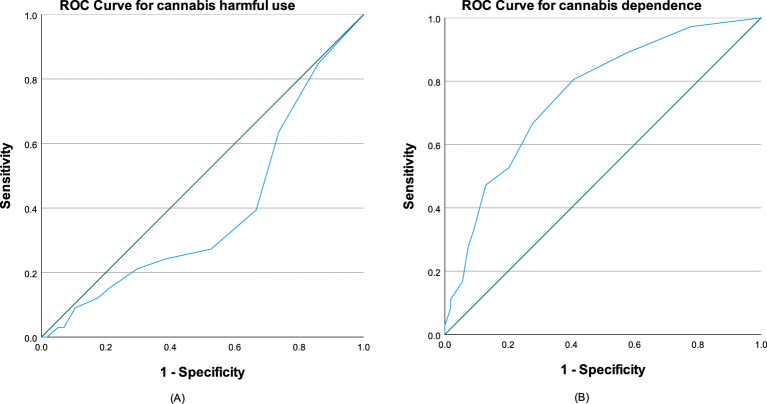
ROC curves of C-SDS-C for **(A)** mild CUD (harmful use) with AUC = 0.391 and **(B)** moderate to severe CUD (dependence syndrome) with AUC = 0.754. The diagonal segments were produced by ties.

The AUC for users with mild CUD (harmful use) was 0.391 (95% CI 0.269 to 0.513), whereas for users with moderate-to-severe CUD (dependence syndrome) the AUC was 0.754 (95% CI 0.657 to 0.858) (**Figure 1**). A cut-off score of ≥ 3 on the C-SDS-C yielded the highest Youden Index, with the sensitivity of 80.6% and specificity of 59.3% (Youden Index = 0.4) (**Table 4**). These findings suggest that the C-SDS-C has significant diagnostic utility for identifying moderate-to-severe CUD but not for mild CUD. Cannabis users scoring 3 points or higher on C-SDS-C are likely to suffer from moderate-to-severe CUD.

**Table 4:** Criterion validity of C-SDS-C at each successive cut-off score on the optimal discrimination for moderate to severity CUD (cannabis dependence syndrome) in cannabis users (N=36).

Score	Sensitivity	Specificity	Youden Index
≥ 1	97.2	22.2	0.19
≥ 2	88.9	42.6	0.32
**≥ 3**	**80.6**	**59.3**	**0.4**
≥ 4	66.7	72.2	0.39
≥ 5	52.8	79.6	0.32
≥ 6	47.2	87	0.34
≥ 7	33.3	90.7	0.24
≥ 8	27.8	92.7	0.2
≥ 9	16.7	94.4	0.11
≥ 10	11.1	98.1	0.01
≥ 11	8.3	98.1	0.01
≥ 12	2.8	100	0
≥ 13	0	100	0
≥ 14	0	100	0

C-SDS-C, Chinese version of the Severity of Dependence Scale for Cannabis; CUD, cannabis use disorder; N, number of participants. Bold value represents the greatest Youden Index.

## 4 Discussion

The current study established the diagnostic utility of C-SDS-C as a valid and reliable screening instrument for moderate-to-severe CUD among Chinese-speaking cannabis users. We demonstrated that all five items in the C-SDS-C were consistent in measuring psychological dependence on cannabis use. The single-factor structure of the C-SDS-C aligns with previous studies that have identified a unidimensional structure of the SDS across various languages and across different substances (17, 21, 22, 25, 30–32).

The ROC analysis in this study suggested that the C-SDS-C possesses adequate discriminative power for moderate-to-severe CUD. The diagnostic cut-off of ≥ 3 in the current study has improved sensitivity in distinguishing cases of cannabis dependence from non-dependence as compared to that identified in earlier study by Swift et al. (23). However, other studies have reported slightly different cut-off scores, such as a lower score of 2 for cannabis users with psychosis (20) and a higher score of 4 for frequent cannabis users in the Netherlands (21) and Canada (33), and adolescent populations (22). In fact, cannabis use and CUD has a bidirectional relationship with significant genetic correlations (34, 35). Levey et al. has recently demonstrated that the genome-wide significant loci for CUD in East Asians were different from those in European, African and Admixed American ancestries (36). Such genetic variabilities could result in different single nucleotide polymorphism-based heritability, fetal and adult frontal cortex gene expression, and potentially resulting in diverse trajectories to the development of CUD. These variations suggest that different cut-off scores may be necessary especially for different populations across ancestries.

Our study demonstrates the ability of the C-SDS-C to identify 80% of moderate-to-severe CUD cases in Chinese-speaking population. Nonetheless, this study did not establish the diagnostic utility of the C-SDS-C for identifying mild CUD among Chinese users. The low prevalence of mild CUD (36.6%) in our sample may have affected the psychometric properties of the C-SDS-C in detecting the latter diagnosis. Despite the recent significant increase in reported cannabis users in Hong Kong (4), only 1.17% of patients with CUD have been admitted to the local hospital Accident & Emergency department over the past decade, which is the second lowest rate among all substance use disorders (12). This may potentially inflate the prevalence of more severe CUD cases within the Hong Kong medical system, from which our participants were recruited. Therefore, the C-SDS-C is suitable for detecting moderate-to-severe CUD specifically within the Chinese-speaking population.

Despite the significant upsurging of CUD and its associated burdens in East and Southeast Asia (37, 38), the proportion of cannabis users receiving treatment in these regions remains the second lowest among all continents (1). In Western countries, low treatment rates for CUD have been attributed to factors such as self-reliance, absence of self-perceived treatment needs, and stigma (21, 39). These issues may be exacerbated in Eastern countries because of collectivistic and assertive attitudes toward cannabis use in Eastern cultures (40, 41). With the ongoing legalization of medical cannabis and decriminalization of cannabis in Asia (3, 42), along with supportive attitudes toward medical cannabis use among Asian medical students (43), it is likely that the prevalence of cannabis use will continue to rise. A recent survey in Taiwan revealed that respondents who supported cannabis legalization were less informed about accurate information regarding cannabis and perceived the impacts of legalization as less important (44). This indicates that awareness of the harms associated with cannabis and education about the consequences of legalization remain insufficient, posting a threat of further increases in cannabis use among Chinese-speaking populations. Therefore, the C-SDS-C will be valuable for the early detection of moderate-to-severe CUD among the Chinese-speaking users. As noted, any diagnostic cut-off involves a trade-off between sensitivity and specificity. To optimize the C-SDS-C as an effective screening instrument, it may be preferable to prioritize higher sensitivity, even at the expense of some specificity, to maximize its ability to detect cannabis users at risk of moderate-to-severe CUD. This approach ensures that cannabis dependent users can receive timely intervention, thereby reduce the subsequent risks to their physical and mental wellnesses.

Our study concurred with three other studies focusing on the Chinese population that Item 4 of the SDS had the weakest factor loading in distinguishing between dependence and non-dependence states (29, 31, 32). Considering the cultural dimension, collectivism in Asian countries prioritizes the needs and goals of family and community over individual desires. Consequently, substance users might avoid seeking help to prevent bringing troubles or shame to their families (41). In addition, substance users within the Chinese population often have heightened concerns about maintaining their moral face and social position, which can lead to greater stigma internalization and increased feelings of shame and guilt (45). Being diagnosed with CUD that requires treatment may be perceived as a moral failure, resulting in a lower desire to stop despite awareness of cannabis dependence. As a result, Item 4 may not be as indicative as other items in assessing the severity of CUD in the Chinese-speaking population.

One major limitation of the current study is its retrospective nature that precluded the assessment of test-retest reliability for the C-SDS-C. Secondly, the cannabis users included in this study mainly resided in Hong Kong and it might limit the generalizability of the C-SDS-C to other Chinese-speaking communities in Western countries. Lastly, differences in treatment accessibility and attitudes towards cannabis use, CUD, or cannabis treatment may limit the extrapolation of the findings to other Asia and Southeast Asia countries. Further validation studies are required to determine whether similar cut-off scores and factor structures apply when using the C-SDS-C in countries with non-Chinese dominant cultures.

## 5 Conclusion

The current study supports the use of the C-SDS-C as a reliable and valid assessment tool for screening moderate-to-severe CUD in Chinese-speaking population, with a recommended cut-off score of 4. Although this study did not demonstrate diagnostic utility of the C-SDS-C in identifying mild CUD, future research should explore its potential to differentiate mild and moderate-to-severe CUD. Additionally, revising the item 4 of the scale may enhance its accuracy for screening purposes in Chinese-speaking cannabis users.

## Data Availability

The data analyzed in this study is subject to the following licenses/restrictions: The data that support the findings of this study are available from the corresponding author upon reasonable request. Requests to access these datasets should be directed to AC, chungkka@hku.hk.
